# Foraging with your eyes: a novel task to study cognitive strategies involved in (visual) foraging behaviour

**DOI:** 10.1007/s10339-025-01261-0

**Published:** 2025-02-21

**Authors:** Matthew Green, Vladislava Segen, Amanda Korstjens, Andrew Isaac Meso, Tessa Thomas, Jan M. Wiener

**Affiliations:** 1 Department of Psychology, Bournemouth University, Fern Barrow, Poole, BH12 5BB UK; 2 Aging & Cognition Research Group, German Center for Neurodegenerative Diseases (DZNE), Leipziger Str. 44, 39120 Magdeburg, Germany; 3 Department of Life & Environmental Sciences, Bournemouth University, Fern Barrow, Poole, BH12 5BB UK; 4 Neuroimaging Department, Institute of Psychiatry, Psychology & Neuroscience (Ioppn), De Crespigny Park, London, SE5 8AF UK

**Keywords:** Foraging, Search, Visual search, Resource distribution, Eye-tracking

## Abstract

In this study we introduce a new gaze-contingent visual foraging task in which participants searched through an environment by looking at trees displayed on a computer screen. If the looked-at tree contained a fruit item, the item became visible and was collected. In each trial, the participant’s task was to forage for a defined number of fruit items. In two experiments, fruit items were either randomly distributed about the trees (dispersed condition) or organised in one large patch (patchy condition). In the second experiment, we addressed the role of memory for foraging by including a condition that did not require memorising which trees had already been visited by changing their appearance (tree fading). Foraging performance was superior in the patchy as compared to the dispersed condition and benefited from tree-fading. In addition, with further analyses on search behaviour, these results suggest (1) that participants were sensitive to the distribution of resources, (2) that they adapted their search/foraging strategy accordingly, and (3) that foraging behaviour is in line with predictions derived from foraging theories, specifically area-restricted search, developed for large scale spatial foraging. We therefore argue that the visual search task presented shares characteristics and cognitive mechanisms involved in successful large-scale search and foraging behaviour and can therefore be successfully employed to study these mechanisms.

## Introduction

Optimal navigation to maximise nutritional return while minimising travel, time and energetic costs is a strong driver in cognitive evolution in humans and other animals, forming a major part of the Ecological Intelligence Hypothesis (Rosati 2017). Every day individuals have to find sufficient food and fluids as well as the right nutrients to satisfy their daily requirements. Effective and efficient foraging requires recognising and responding to the distribution of resources in the environment (Senft et al. 1987) and the ability of the forager to avoid revisiting empty resources (Viswanathan et al. 2011). Foraging theories state that foragers should adapt their search behaviour according to distribution of resources in the environment in terms of abundance and patchiness in space and time (Senft et al. 1987), their knowledge of the location of resources in the environment, and whether resources are depletable (Emlen 1966; Janson 2019; MacArthur and Pianka 1966; Pyke 1984).

Traditionally, foraging theories have been concerned with explaining locomotion and navigation behaviour in large scale environments (e.g., spatial foraging). Recent research, however, has demonstrated that searching through small two-dimensional (2D) spaces (e.g., pictures) or even internal spaces (e.g., memory) relies on similar characteristics and shares neural as well as cognitive mechanisms with searching in large spaces (for an overview, see Hills 2006; Hills et al. 2015). This provides opportunities for studying foraging theory using paradigms that make use of 2D spaces.

Visual foraging tasks in 2D spaces have been used primarily to study the marginal value theorem stating that foragers should exploit patches rich in resources until resource retrieval rates fall below the global retrieval rate (Charnov 1976). Once this threshold is reached, foragers should switch from exploitation to exploration to find another resource patch to exploit (Charnov 1976). Wolfe (2013), for example, used a laboratory analogue of a berry picking task to study if humans are sensitive to rates of resource intake in visual foraging and to study patch leaving behaviour. In the task, participants were presented with visual displays of “berries” and “leaves” and picked berries by clicking on them. When they picked as many berries as desired, they could move to a new patch. Results demonstrate that humans foraging or searching through 2D displays are sensitive to rates of intake and that their behaviour can be modelled with variants of Charnov’s Marginal Value Theorem (see also Zhang et al. 2017).

The marginal value theorem models optimal patch departure time, but it does not explain how foragers efficiently exploit resource patches, especially in situations in which patch boundaries cannot be detected by foragers. Area restricted search (or area-concentrated search) is a common search pattern that can account for switching back and forth between exploration and exploitation (for a recent review, see Dorfman et al. 2022). Specifically, organisms respond to resource encounters by transitioning to a local intensive search and back to exploratory behaviour when resource encounters decrease. In environments with patchy resource distribution such behaviour increases the likelihood of encountering further resources, thus helping to efficiently exploit resources in environments characterised by patchy resource distributions.

While area restricted search has been described in many animal species (Dorfman et al. 2022), there currently are only a few human studies. Hills et al. (2013), for example, asked participants to search for hidden resources in open-field virtual environments and showed an increased turning response after resource encounters in environments with patchy resource distributions but not in environments in which resources are dispersed. This behaviour which is characteristic of area-restricted search has also been observed when searching for hidden resources in small two-dimensional spaces presented on a computer screen further highlighting that foraging or search in simple two-dimensional spaces relies on similar characteristics as search in large spaces (Hills 2006).

### The current study

The overall aim of this study was to develop a new visual foraging task that shares characteristics and cognitive mechanisms involved in successful large-scale search and foraging behaviour. Specifically, our task was designed to study how human foragers respond to resource encounters and how they adapt their search strategy when searching environments which have patchy or dispersed resource distributions. We decided to use eye-tracking and gaze contingent procedures, which provide a continuous window into the allocation of (overt) attention over the two-dimensional search space presented (Hollingworth and Bahle 2019). While eye movements are not a direct measure of cognitive processes, there is often a close relationship between where people are looking and what they are thinking about (the eye-mind hypothesis of Just and Carpenter 1980). Analyses of eye-movements can therefore provide additional information about higher order cognitive processes involved in visual search (Johannesson et al. 2016). For example, at the sufficiently high resolution of 1000 Hz, that we employ in the current study, eye movements tap into a quick and low noise readout of sensorimotor circuits in the thalamus, visual and prefrontal cortex associated with attention and decision making (Martin et al. 2018; Meso et al. 2016; Schütz et al. 2012).

In the experiments, participants were asked to ‘forage’ for a set number of resources that were hidden in trees. Specifically, participants visually inspected trees that were displayed on a computer screen. If a tree contained a hidden target (i.e. fruit or resource item), the target appeared and was collected as soon as the participant’s gaze landed on that tree. This task design shares some common features with classic visual search paradigms particularly those carried out while eye movements were recorded. During foraging periods, memory may therefore play a role of increasing efficiency (Dickinson and Zelinksy 2007) and shaping the scan path, or alternatively, searches may tend to follow systematic paths (Gilchrist and Harvey 2006). Whether or not participants tend to return to previously searched locations can also be interpreted in terms of the studied and debated phenomenon of Inhibition of Return (Klein and MacInnes 1999; Hooge et al. 2005). While we focus primarily on the foraging aspects, we recognise that participant behaviour will also be shaped by cognitive constraints on visual search and revisit these in the discussion.

In Experiment 1, participants were asked to find 10 out of the 15 fruit items that were hidden among 30 trees. In the dispersed condition, these fruit or target items were randomly distributed about the 30 trees. In the patchy condition the target items were organised in one large patch that could either be on the left or right side of the screen. We predicted that participants would be sensitive to the resource distribution and would adapt their foraging strategy accordingly (Hills et al. 2013). In line with area restricted search, we thus expected that following resource encounters in the patchy condition, participants would transition to intensive search (Hills et al. 2013). This should lead to superior foraging success in the patchy as compared to the distributed condition. Finally, we expected to see differences in the global search patterns between the dispersed and the patchy condition and evidence for the switch between exploitation and exploration in the patchy but not in the dispersed condition (Dorfman et al. 2022).

The aim of Experiment 2 was twofold. The first aim was to provide a conceptual replication of Experiment 1. The second aim was to investigate the role of task difficulty in visual foraging behaviour. In addition to manipulating resource distribution (as in Experiment 1), we manipulated the difficulty of the task by requiring participants to find 14 out of the 15 fruit items (instead of 10/15 as in Experiment 1) and by introducing a condition in which trees that had been looked at changed their appearance to indicate that they had been visited and did not contain target/fruit items. We expected participants to show more efficient foraging behaviour (i.e., shorter time spent exploring and fewer mistakes) when they can easily identify which resources have been visited. We also expected that the benefit of visually marking trees that were already visited would be most pronounced when food resources were dispersed randomly rather than patchily. This is because the patchy distribution should allow foragers to effectively guide search to a smaller part of the search space.

## Experiment one

### Methods

#### Participants

47 participants took part in the experiment (but 5 of those did not yield data because of technical issues during the experiment, leaving 42 participants with a mean age of 25.5 years (age range 20–48 years; s.d.6.3 years; 29 females and 13 males). Participants received course credits or monetary compensation for their participation. The experiment was part of a larger battery of five short experiments, each of which could be broadly described as a ‘visual cognition’ eye-tracking task: however, this task was the only ‘foraging’ task in the battery and we do not report results of the other experiments here. The five tasks were always presented in the same order with the foraging task being the last task. The total duration of the battery of five experiments was about one hour including calibration and rest breaks, and the task described here took participants less than 10 min to complete, taking advantage of rapid data collection possible from eye movement tasks.

#### Ethics

Ethical approval was obtained from the Science, Technology & Health Research Ethics Panel at Bournemouth University and written informed consent was obtained from all participants, in accordance with the Declaration of Helsinki (World Medical Association 2001).

#### The foraging task

**Fig. 1:**
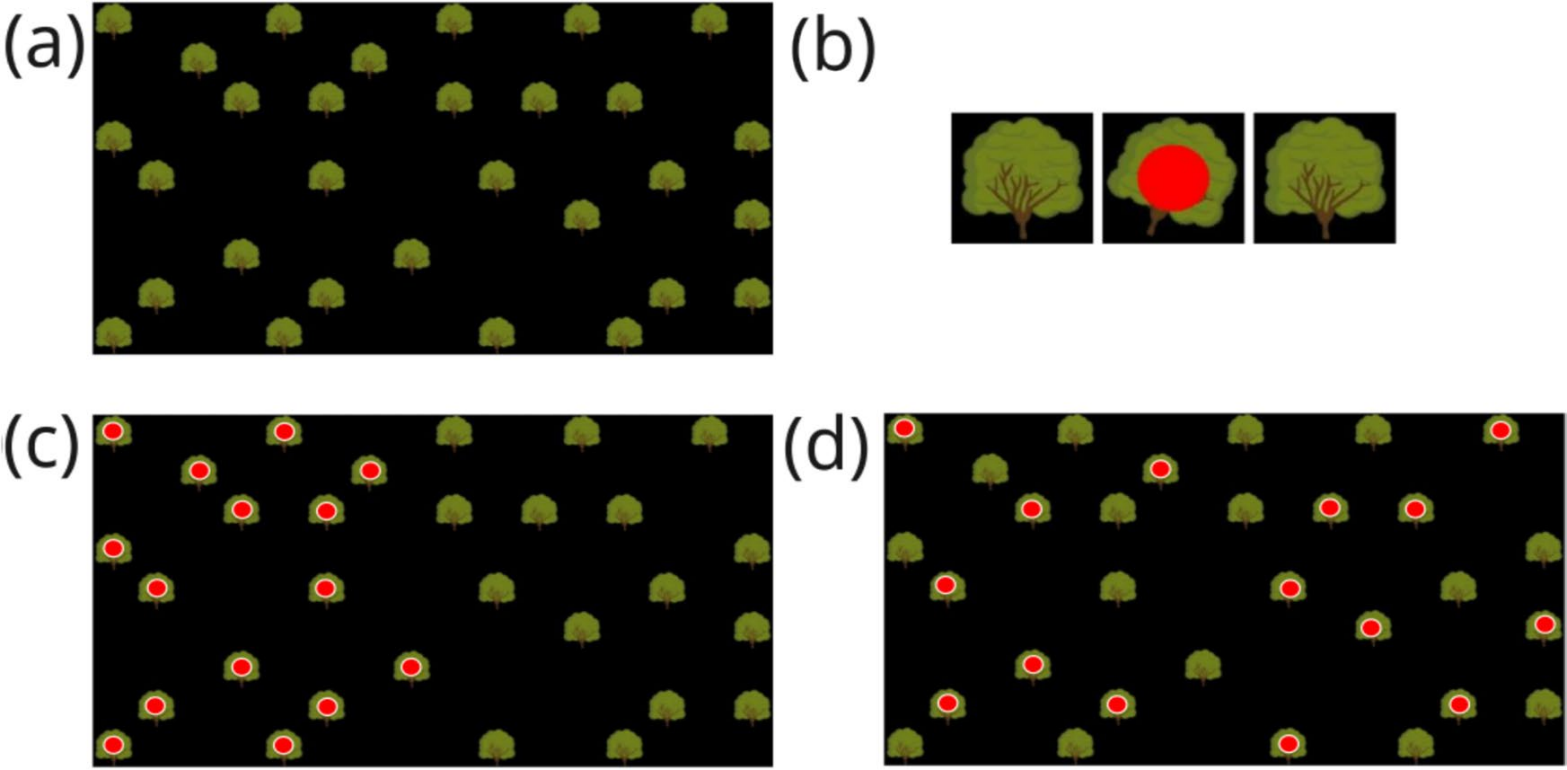
(**a**) Example layout of trees; (**b**) sequence showing the gaze-contingent display change when participants focused on a tree (i.e., when they fixated a tree). The tree tilted and if it carried a fruit item it was revealed and indicated by a red dot: if the tree did not contain a fruit then no red dot was presented (**c**) & (**d**) Examples of the distribution of the fruits over the trees for the patchy condition (c) and the dispersed condition (**d**). Panel (**c**) shows a trial in which the resource patch was on the left side, but there was an equal number of trials in which the patch was on the right side of the display. Note that the fruit items (red dots) in panels (**c**) and (**d**) are superimposed on the trees and accentuated by a white border for illustrative purposes only. Participants never saw the fruit/target items superimposed on the trees unless they focused on a tree that carried a fruit item. Only that fruit item was then revealed as shown in Panel (**b**)

The computerised gaze contingent task consisted of 20 individual trials. In each trial participants were presented with a display containing 30 trees (see Fig. 1), 15 of which contained a hidden fruit item which was the target (an apple, represented by a filled red circle). On each trial, the participant’s task was to forage for and retrieve 10 of the 15 fruit items. They did so by directing their gaze towards a tree. The eye-tracker sampling rate was 1000 Hz and if a sample of gaze (i.e., one millisecond of gaze duration) was directed at a tree, the tree gave visual feedback (described in detail below) that generated a strong visual onset on the fovea indicating to the participant that the software had detected the participant's gaze on that tree.

The visual feedback consisted of oscillation of the tree; and the appearance of a red dot if the tree contained fruit (see Fig. 1). The oscillation was between two states: the states were (1) tilted at 22.5 degrees for 83 ms and (2) upright for 8.3 ms. If the tree contained fruit then a red dot was overlaid on the first tilted phase, i.e., for 83 ms. When gaze left the tree the oscillation stopped after completing the current cycle of tilted / upright.

Importantly, the procedure for generating oscillation in response to gaze was highly constrained in order to avoid accidental oscillation. It was not possible for multiple trees to oscillate simultaneously. Thus, in a situation where tree A received a sample of gaze and started to oscillate, and if tree B received a sample of gaze before tree A had finished oscillating, tree B would not be free to oscillate until tree A had finished oscillating, and even when tree A had finished oscillating, tree B would only oscillate if tree B was still receiving gaze samples at the point where tree A had finished oscillating. These constraints combined to make it extremely unlikely that tree B would be caused to oscillate by any sample of gaze on tree B that was received during a saccade passing through tree B on its way from tree A to tree C.

If a fruit-bearing tree was revisited after its fruit had been foraged then no fruit would appear but the tree would still oscillate.

To retrieve 10 fruit items, participants therefore had to move their gaze across the display and land on different trees. A single trial was terminated as soon as participants retrieved the 10th fruit item or after 30 s had elapsed.

#### Stimuli

We designed 20 stimuli with different tree layouts. To do so, we created a nonvisible rectangular grid of squares with 16 columns and 9 rows. Thirty trees were pseudo-randomly distributed about the grid such that two horizontally and vertically neighbouring grid cells were not both occupied by trees (see Fig. 1), i.e. trees never overlapped. Each tree measured 108 pixels in both width and height, corresponding to a visual angle of approximately 2.80° vertically and 2.68° horizontally. The average closest distance between trees was 232.97 pixels (approximately 5.77° of visual angle), whilst the average distance between all trees was 858.86 pixels (approximately 21.27° of visual angle). The resources, i.e. the target fruit items, were then distributed about the trees in either a dispersed or patchy fashion. We created ten dispersed stimuli in which the 15 target fruit items were randomly distributed in advance over the 30 trees (dispersed condition) and ten stimuli in which all 15 target fruit items were arranged in one large patch (patchy condition) that covered either the left or the right side of the layout (see Fig. 1). All stimuli were prepared in advance and each participant saw the same stimuli (i.e., tree positions were not generated on the fly at the start of every trial).

#### Apparatus

Eye movements were captured using an EyeLink 1000 tower mount (EyeLink 1000, SR Research Ltd., Ottawa, Canada) sampling the pupil position of the right eye at 1000 Hz (if there were problems calibrating the right eye, the left eye was tracked instead – for example, one participant had a corneal scar on the right eye so that it was only possible to track the left eye). Calibration was performed and checked for accuracy before starting the experiment using a nine-point grid. Drift correction was performed before each stimulus presentation. We used custom made software programmed in Python using libraries from PsychoPy (Peirce et al. 2019) to display and update stimuli in response to the current gaze position combined with the history of gaze positions at that point in the trial (allowing us to display fruit only on the first viewing and not on subsequent viewings, for example), and to record a time series of (x, y) gaze positions for later analysis. The experiment was presented on an 80.9 cm (diagonal) screen with a width of 70.5 cm and a height of 39.7 cm, featuring a 16:9 aspect ratio and a resolution of 1920 × 1080 pixels with a 120 Hz refresh rate. Participants were seated 80 cm from the monitor with their head positioned on a chin rest. The physical vertical field of view (FOV) of the screen at this distance was 28°, and the horizontal FOV was 47.7°

#### Procedure and design

The experiment was run in a small room with minimal distraction. Participants were instructed about the nature of the experiment and signed the consent form. After calibration they were given two practice trials with fewer trees to explain how to interact with the experiment. Specifically, they were first shown a display with only eight trees and asked to direct their gaze towards one tree to demonstrate how gazing at a tree would trigger the tree to shake. They were then asked to direct their gaze to a second tree which also oscillated and revealed a target fruit item. Once participants understood how to interact with the experiment and after it was explained to them that they had to search for and collect ten target fruit items they were given another practice trial with 12 trees before the actual experiment began.

In Experiment 1 participants completed two blocks of ten trials. One block contained all the patchy stimuli (patchy condition), the other block contained all the dispersed stimuli (dispersed condition). The order of blocks, the order of the stimuli in each condition as well as the side of the patch in the patchy distribution was randomised at runtime for each participant. Participants were not informed about the differences in resource distribution.

#### Analysis

We calculated and analysed the following four metrics:

1. Number of Trees: we recorded the total number of trees (including revisited trees in the count) that participants directed their gaze at on each trial. This is the primary measure of foraging performance.
2. Revisits: the number of times participants revisited a tree that they had already looked at. We consider these revisits as memory errors.
3. Retrieval rate: describes the number of trees participants visited to retrieve one fruit/resource item. A low number of trees visited per fruit item indicates a high retrieval rate and therefore more efficient foraging.
4. Inter-tree distance: describes the distance in degrees of visual angle between two successively visited trees. To investigate search strategy, we specifically compared inter-tree distance depending on whether the tree from which the eye-movement started (later referred to as the ‘launch site’ of that eye-movement) did or did not contain a fruit / target. Since the patchy and dispersed trials were blocked, participants in the patchy condition could learn over trials that all fruit items are on the same side of the tree layout. If they then encounter a tree without a fruit item, they could reasonably assume that they are in the wrong patch (patch without fruit), and then make a large movement to the other side of the screen.

In addition, we binned the trials into early trials (trials 1–5) and late trials (trials 6–10) for the analyses of the number of trees participants visited per trial, and number of revisits per trial. Binning the trials into early and later phases allowed us to effectively analyse learning patterns. Early trials represent the initial phase of exposure, while later trials reflect the participants' adaptation to the specific resource distribution.

Five of the 47 participants were excluded from the final data set because the experiment crashed during their session and no data was recorded, leaving 42 participants. We then discarded individual trials during which participants did not collect at least 10 fruit items before the trial timed out after 30 s. This resulted in 23 out of 840 trials (i.e., 2.74%) being removed, leaving 817 trials in the final data set for analysis.

### Results

#### Number of trees

**Fig. 2:**
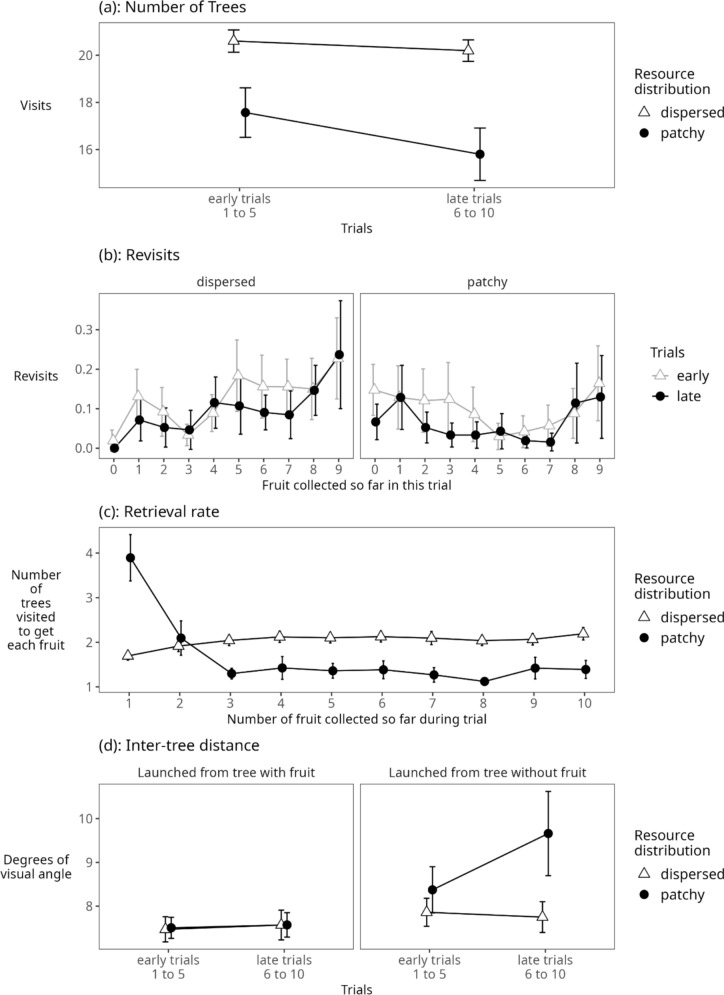
(**a**) Total number of trees visited in the dispersed and patchy condition in early and late trials; (**b**) Average number of memory errors (revisits) in the dispersed and patchy condition in early and late trials; (**c**) Number of trees visited for each fruit/target item over the course of a trial; (**d**) Distance between successively visited trees after fruit encounter (left) or after visiting tree without fruit (right)

A 2 × 2 ANOVA with the within factors ‘resource distribution’ (patchy, dispersed) and ‘trial’ (early [mean of trials 1 to 5], late [mean of trials 6 to10]) revealed main effects of resource distribution (F (1, 41) = 52.17, *p* < 0.001) and trial (F (1, 41) = 17.43, *p* < 0.001) on the number of trees visited. Specifically, participants visited fewer trees to complete the trial in the patchy condition than in the dispersed condition (16.69 ± 3.06 vs 20.40 ± 1.26), and participants visited fewer trees on average in late than in early trials (early: 19.09 ± 1.84; late trials 18.00 ± 1.85). The interaction was also significant (F (1, 41) = 5.04, *p* = 0.03), highlighting that the number of trees visited to complete a trial declined more in the patchy than the dispersed condition over the course of the experiment (Fig. 2a).

#### Number of revisits

A 2 × 2x10 ANOVA with the factors ‘resource distribution’ (patchy, dispersed) and ‘trial’ (early [mean of trials 1–5], late [mean of trials 6–10]) and number of fruit consumed (1–10) revealed significant main effects of trial (F (1, 41) = 17.83, *p* < 0.001) and number of fruit consumed (F (4.37, 177.5) = 6.39, *p* < 0.001, after Greenhouse–Geisser correction for sphericity violation) but not of resource distribution (F (1, 41) = 3.84, *p* = 0.06). Specifically, participants revisited trees significantly less often in late trials than in early trials (late 0.08 ± 0.07; early 0.11 ± 0.08) and they revisited trees more frequently towards the end of a single trial than at the beginning of a trial (mean revisits for the first fruit collected: 0.06 ± 0.06; mean revisits for the last fruit collected: 0.19 ± 0.21). Only the interaction resource distribution x number of fruit was significant (F (4.5, 182.6) = 4.41, *p* < 0.01 after Greenhouse–Geisser correction for sphericity violation; all other interactions: *p* > 0.05 (Fig. 2b).

#### Retrieval rate

A 2 × 10 ANOVA with the within factors ‘resource distribution’ (patchy, dispersed) and ‘trial’ (early [mean of trials 1–5], late [mean of trials 6–10]) and number of fruit consumed (1–10) revealed a significant main effects of resource distribution (F (1, 41) = 54.14 *p* < 0.001) and number of fruit consumed (F (3.91, 160.43) = 22.99, *p* < 0.001 after Greenhouse–Geisser sphericity correction), as well as a significant interaction (F (4.83, 197.91) = 46.85, *p* < 0.001 after Greenhouse–Geisser sphericity correction). Participants visited fewer trees to retrieve fruit items in the patchy condition as compared to the dispersed condition (patchy 1.67 ± 0.30 vs dispersed 2.04 ± 0.13). Participants needed to visit fewer trees to retrieve fruit items later in the trial (mean at the point where one fruit had been consumed was 2.79 ± 0.90; mean for the point at which ten fruits had been consumed was 1.79 ± 0.38). Figure 2c shows the nature of the interaction: specifically, the number of trees visited to retrieve fruit items decreased the more fruits were consumed in the patchy condition while it stayed consistently high (∼2 trees per fruit item) as more fruits were consumed in the dispersed condition.

#### Inter-tree distance

A 2 × 2 × 2 ANOVA with the within factors ‘resource distribution’ (patchy, dispersed) and ‘trial’ (early [mean of trials 1–5], late [mean of trials 6–10]) and ‘launch site’ (i.e., whether a movement was launched from a tree containing fruit or from a tree that did not contain fruit) revealed significant main effects of resource distribution (F (1, 41) = 13.09, *p* < 0.01), trial (F (1, 41) = 6.18, *p* < 0.05) and launch site (F (1, 41) = 31.62, *p* < 0.001). The distance between subsequently visited trees increased significantly (Fig. 2d) between early and late trials. This main effect of trial was driven by a three-way interaction between resource distribution, trial and launch site (F (1, 41) = 11.62, *p* < 0.01). Specifically, inter-tree distance increased between early and late trials only for the patchy condition and only if the last visited tree did not contain a fruit item (from 8.4 ± 1.7 to 9.7 ± 3.1 degrees of visual angle; see Fig. 2d). In all other cases, the travel distance remained relatively unaffected between early and late trials.

#### Scanpaths

**Fig. 3:**
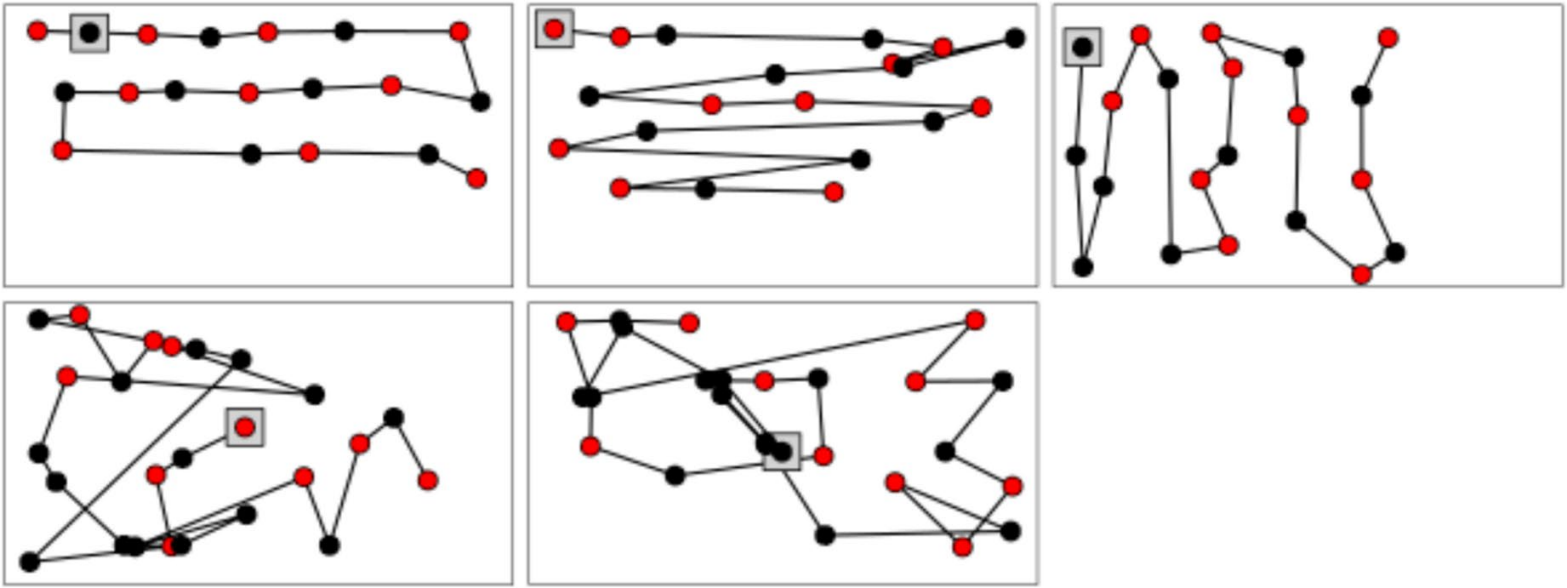
Examples of foraging scanpaths from the dispersed condition. Points indicate fixations in trees. Points for trees bearing fruit have red centres: points for trees not bearing fruit have black centres. A square point indicates the first fixation in the trial. The top row shows some systematic search patterns, and the bottom row shows some examples of fairly chaotic search behaviour. Top-left: horizontal lawnmower, regular movements between trees; Top-middle: a cross between reading and lawnmower: some return-sweep-like movements. Diagonal movements are the main type, whereas there are no diagonal movements in the top-left plot. Top-right: vertical lawnmower: this one is interesting because it is not inherited from reading – the movements are up and down instead of left to right. Bottom-row: participants did not follow a clear search pattern but seem to be able to remember previously visited trees considering that there were few revisits

Figure 3 presents examples of foraging scanpaths. While we will not aim to classify different search strategies here, it is apparent that the scanpaths in the top row were very systematic. These kinds of search patterns can result from strategies referred to as grid-like or lawn mower strategies (e.g., Tellevik 1992; Gilchrist and Harvey 2000) allowing to search a space without oversampling (i.e., revisiting already visited sites). The scanpaths in the bottom row were more irregular and included revisits.

### Discussion

Our results show that participants were sensitive to the resource distribution (c.f. Hills et al. 2013). Participants performed better in the patchy than the dispersed condition, i.e., they searched fewer trees to find the required resources. Even though not reaching statistical significance (*p* = 0.06), they also made fewer errors, that is, they revisited trees less frequently. In the dispersed condition participants inspected just over 20 trees to collect 10 fruit items throughout the experiment. This was expected as every other tree contained a fruit item, which were randomly distributed about the trees. In the patchy condition, in contrast, participants’ performance increased over the course of the experiment and in the second half of the experiment they only inspected 16 trees. This success rate was higher than chance level, and demonstrates that participants learned, over the course of the experiment, that the resources were organised in patches, even though the patch could either be on the left or the right side of the screen. Importantly, it also shows that participants adapted their search/foraging strategy to the resource distribution over the course of the experiment.

As participants’ performances in the patchy condition increased, so did the travel distance between trees after unsuccessful tree visits. This result suggests that participants understood that they were in a part of the environment that did not contain a resource patch and needed to explore a different more distant part of the environment to find the resource patch. This interpretation is also in line with the retrieval rate results from the patchy condition that show that within a single trial, participants initially needed to search multiple trees to find the first fruit items. Once these were found, that is, once the resource patch was found, retrieval rate increased, and participants very efficiently exploited the patch. For the rest of the trial, they showed almost perfect retrieval, that is, they found a fruit item almost every time they looked at a tree. These results are consistent with the concept of area restricted search where foragers switch from exploration to exploitation when encountering ‘hidden’ resource items in environments with patchy resource distribution (Dorfman et al. 2022). We are therefore confident that the visual foraging task presented here is capturing important aspects of search/foraging behaviour studied in real environments (Hills et al. 2015).

During the experiment, participants revisited trees they had visited before. Given that fruits did not replenish during a trial, revisits are suboptimal behaviour or errors indicating that participants did not remember that they had visited the trees before. The number of errors declined over the course of the experiment, suggesting that participants adapted their search behaviour as they got more familiar with the task. Not surprisingly, memory errors increased towards the end of a trial, when participants searched for the last fruit items and only few fruit items remained in the environment. While not reaching statistical significance (*p* = 0.06), participants produced fewer memory errors in the patchy than the dispersed condition, likely because understanding that resources were organised in a large patch allowed them to reduce the effective search space which made memorising which trees had been visited easier.

Overall, there were few revisits, suggesting that (1) the task of finding 10 of the 15 fruit items hidden in the environment was relatively easy and/or (2) that participants employed search/foraging strategies to search the environment while avoiding oversampling. Visual inspection of selected search paths suggests that participants, in some cases, employed very systematic search patterns that could be described as grid-like or lawn mower strategies (Tellevik 1992) while other cases suggest less strategic search strategies. However, given the difficulty of quantifying and characterising search strategies based on trajectories alone (Kembro et al. 2019), we will address this aim in future research.

## Experiment two

**Fig. 4:**
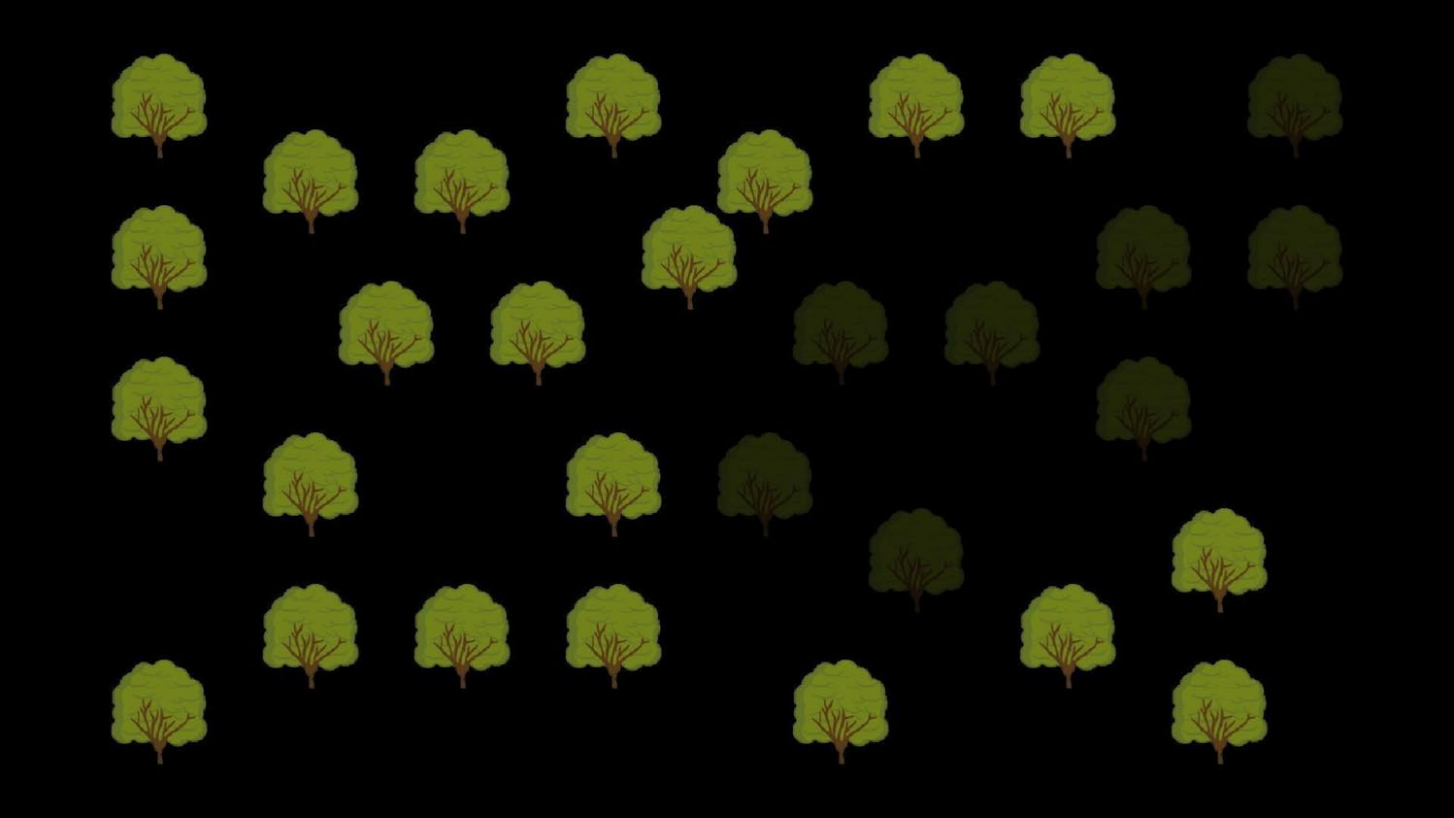
An example of the visual display during a trial in which some trees that had already been visited are faded out to aid memory of which trees were visited

The aims of Experiment 2 were (1) to provide a conceptual replication of Experiment 1, and (2) to further investigate the role of (spatial) memory in our foraging task. To address the second aim, we changed the experiment as follows: First, we increased the difficulty of the task by asking participants to find 14 out of the 15 fruit items in each trial. Second, we introduced a manipulation in which trees that have been visited either changed appearance (fade out, see Fig. 4) or remained as they were. The latter condition is the same as in Experiment 1. In the tree fading condition, in contrast, participants did not need to memorise the locations that have already been visited.

We expected to replicate findings from Experiment 1. That is, we expected better performance in the patchy than the dispersed condition and that this difference increased over trials. If the low re-visit rates in Experiment 1 could be explained by participants’ use of very systematic search strategies such as a ‘lawn mower’ strategy (see Fig. 3), we expected the tree fading to have little or no effect on foraging performance. If, on the other hand, the low re-visit rates in Experiment 1 mainly reflected that the task was relatively easy, we expected that the tree fading manipulation would affect foraging performance. Specifically, participants should show fewer errors (revisits) in the tree-fading condition which does two things: First, it supports memory by visually marking trees that had already been visited. Second, tree-fading reduces the visual saliency of locations/trees that have already been visited which should therefore reduce the likelihood that trees are re-visited. Participants in the no-fade condition, in contrast, should find it harder to find the last few target items, i.e., retrieval rate should decrease as the trial progresses, while revisits (i.e., errors) should increase. Finally, we expect the effect of tree fading to be stronger in the dispersed than the patchy condition.

### Methods

#### Participants

Forty-two participants took part in the experiment (mean age 20.6 years; age range 19–28; s.d.1.9; 34 females and 8 males) and received course credits or monetary compensation for their participation. None of the participants in Experiment Two took part in Experiment One.

#### Foraging task

The foraging task was similar to Experiment 1, but participants had to find 14 out of the 15 available fruit items. Participants completed 40 trials in total (20 patchy; 20 dispersed) instead of 20 trials in total as in Experiment 1.

#### Apparatus

We used the same experimental setup as in Experiment 1, with the exception of a smaller screen and a different eye-tracking system. The screen had a diagonal size of 61.0 cm, with a display area measuring 53.1 cm in width and 29.9 cm in height, and operated at a refresh rate of 60 Hz. Participants were seated at a distance of 80 cm from the screen, resulting in a visual angle of approximately 36.72° horizontally and 21.17° vertically. Additionally, we used an EyeLink 1000 desktop mount instead of the EyeLink 1000 tower mount.

#### Stimuli

The manipulation of resource distribution was implemented within-participants as in Experiment 1, with a block of patchy-only trials and a block of dispersed-only trials, with the order of blocks randomised for each participant. We added a condition in which the trees faded after they had been viewed (see Fig. 4). This was manipulated between participants, so that half the participants had trees that faded, and the other half had trees that did not fade (as in Experiment 1). This manipulation was implemented as a between subjects' factor to avoid order effects. Specifically, we wanted to avoid participants developing a search strategy such as a lawn mower strategy in the no-fade conditions, that then carried over and affected behaviour in the tree-fading condition, which would have reduced the effect of tree-fading. The tree size was 108 pixels in widths and height resulting in 2.07° horizontal and 2.12° visual angle. The average closest distance between trees was 235.83 pixels (approximately 4.51° of visual angle), whilst the average distance between all trees was 866.94 pixels (approximately 16.37° of visual angle).

#### Procedure

Apart from the changes to the stimuli, the procedure was identical to Experiment 1.

### Results

In Experiment 2, *early* refers to trials 1–10 and *late* refers to trials 11–20.

#### Number of trees

**Fig. 5:**
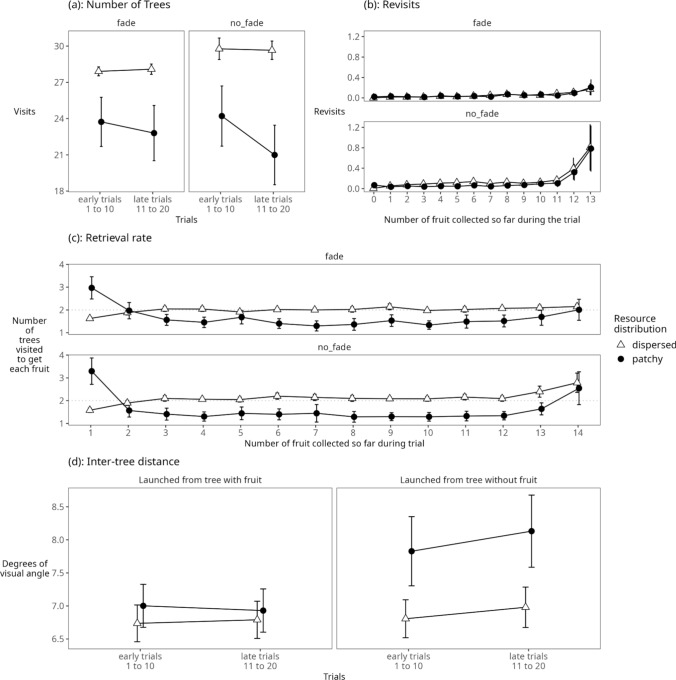
(**a**) Total number of trees visited in the dispersed and patchy condition in the first and second half of the experiment; (**b**) Revisits (memory errors) over the course of a trial in the dispersed and patchy condition and the fade and no fade condition; (**c**) Number of trees visited for each fruit/target item over the course of a trial; (**d**) Distance between successively visited trees after fruit encounter (left) or after visiting tree without fruit (right)

A 2 × 2 × 2 ANOVA with the within factors ‘resource distribution’ (patchy, dispersed) and ‘trial’ (early [mean of trials 1–10], late [mean of trials 11–20]) and the between factor ‘tree fading’ (fading, not fading) revealed significant main effects of resource distribution (F (1, 40) = 71.44, *p* < 0.001) and trial (F (1, 40) = 22.33, *p* < 0.001) but not of tree fading (F (1, 40) = 0.37, *p* = 0.55). As in Experiment 1, participants visited significantly fewer trees in the patchy condition than the dispersed condition (mean patchy: 22.95 ± 4.89 trees; mean dispersed: 28.82 ± 1.51 trees, Fig. 5) and fewer trees in late trials than early trials (mean early: 26.38 ± 2.84; mean late: 25.39 ± 2.91). Of the two-way interactions, tree fading x trial (F (1, 40) = 8.92, *p* = 0.005) and resource distribution x trial (F (1, 40) = 28.22, *p* < 0.001) were significant. The interaction between fading and trial highlights that in early trials of the experiment, participants visited more trees in the no fade condition than in the fade condition, but that in the late trials they visited about the same number of trees regardless of fading. The interaction between trial and resource distribution resulted from participants improving performance (i.e., visiting fewer trees) over trials only in the patchy condition, while performance remained constant in the dispersed condition (see Fig. 5a).

#### Number of revisits

A 2 × 2 × 2 × 14 ANOVA with the within factors ‘resource distribution’ (patchy, dispersed), ‘trial’ (early [mean of trials 1–10], late [mean of trials 11–20]) and ‘number of fruit consumed’ (1–14) and the between factor ‘tree fading’ (fading, not fading) revealed significant main effects of fading (F (1, 40) = 16.97, *p* < 0.001) and number of fruit consumed (F (1.2, 49.6) = 21.63, *p* < 0.001, after Greenhouse–Geisser correction for sphericity violation), but not of resource distribution (F (1, 40) = 2.86, *p* = 0.10) or trial (F (1, 40) = 0.72, *p* = 0.40). When the trees did not fade, the number of revisits was significantly greater (mean for no-fade = 0.15, ± 0.10 revisits) than when trees did fade (mean for fade: 0.05 ± 0.05 revisits). The number of revisits increased over the course of a trial with revisit rates being highest for the last fruit items collected (mean first fruit item: 0.02 ± 0.03; mean last fruit item: 0.48 ± 0.67). There was a significant interaction between fading and number of fruits consumed (F (1.2, 49.6) = 8.87, *p* < 0.01 after Greenhouse–Geisser correction for sphericity violation) which was driven by a stronger increase in revisits towards the end of the trial when trees did not fade (see Fig. 5b). None of the other interactions were significant (all *p* > 0.05).

#### Retrieval rate

A 2 × 2 × 14 ANOVA with ‘resource distribution’ (patchy, dispersed) as a within effect, ‘tree fading’ (fading no fading) as a between effect and ‘number of fruit consumed’ (1–14) as a within effect revealed a significant main effects of resource distribution (F (1, 40) = 71.71, *p* < 0.001) and number of fruit consumed (F (5.3, 211.7) = 17.22, *p* < 0.001, after Greenhouse–Geisser correction for sphericity violation), but no significant main effect of tree fading (F (1, 40) = 0.36, *p* = 0.55). Retrieval rate was better in the patchy than the dispersed condition (mean number of trees visited to get each fruit in the patchy condition: 1.64 ± 0.35; mean dispersed: 2.06 ± 0.11) and retrieval rate was worse at the beginning and end of a trial, i.e., for the first and last fruit item collected during a trial than for the rest of the fruit items. There were two significant interactions, tree fading x number of fruit consumed (F (5.3, 211.7) = 2.89, *p* < 0.05, after Greenhouse–Geisser correction for sphericity violation) and resource distribution x number of fruit consumed (F (6.1, 244.2) = 32.17, *p* < 0.001, after Greenhouse–Geisser correction for sphericity violation). Figure 5c shows the nature of these interactions: importantly at the beginning of the trial the number of trees visited to retrieve fruit items is higher in the patchy than the dispersed condition. The pattern then changes, and the number of trees visited to retrieve a fruit item decreased in the patchy condition while it stayed consistently high (∼ 2 trees per fruit item) as more fruit were consumed in the dispersed condition. At the end of the trial, when participants searched for the last fruit items, the retrieval rates in both conditions then converged. The interaction between tree fading and number of fruits consumed is driven by a lower retrieval rate (more trees visited to collect a fruit item) in the no fade condition for the last fruit item collected.

#### Inter-tree distance

A 2 × 2 × 2 × 2 ANOVA with the within participants factors ‘resource distribution’ (patchy or dispersed), ‘trial’ (early [mean of trials 1–10], late [mean of trials 11–20]) and ‘launch site’ (i.e., whether a movement was launched from a tree containing fruit or from a tree that did not contain fruit) and the between participants factor ‘fading’ (trees fade, trees do not fade) revealed main effects of resource distribution (F (1, 40) = 35.46, *p* < 0.001) and launch site (F (1, 40) = 28.98, *p* < 0.001), but not of early vs late trials (F (1, 40) = 3.64, *p* = 0.06) or fading (F (1, 40) = 0.80, *p* = 0.38). Specifically, the distance between successively visited trees (inter-tree distance) was longer in the patchy condition (mean patchy: 7.5 ± 1.2 degrees of visual angle, Fig. 5d) than in the dispersed condition (mean dispersed: 6.8 ± 0.9 degrees of visual angle) and the inter-tree distance was longer for movements launched from no-fruit trees (7.5 ± 1.2 degrees of visual angle) than for those launched from fruit trees (6.9 ± 0.9 degrees of visual angle). There was a significant interaction between resource distribution and launch site (F (1, 40) = 23.61, *p* < 0.001): in the dispersed condition, the inter-tree distance did not depend on whether the movement launched from a fruit tree (dispersed fruit = 6.8 ± 0.9 degrees of visual angle) or a no-fruit tree (dispersed not-fruit = 6.9 ± 0.9 degrees of visual angle). In the patchy condition, however, the inter-tree distance was longer when the movement was launched from a no-fruit tree (patchy not-fruit 8.0 ± 1.6 degrees of visual angle) than from a fruit tree (patchy fruit 7.0 ± 1.0 degrees of visual angle), likely because participants, after an unsuccessful encounter, understood that they were searching through a part of the environment that did not contain the fruit patch and therefore initiated a longer movement. There was also a significant interaction between launch site and early vs late trials (F (1, 40) = 4.20, *p* < 0.05): while launches from fruit-bearing trees stayed short-distance across trials, launches from non-fruit-bearing trees started short-distance but became longer-distance for late trials, likely reflecting that participants learned about the patches in the environment. None of the other interactions were significant.

### Discussion

The first aim of Experiment 2 was to provide a conceptual replication of results from Experiment 1. Even though we increased the difficulty of the task and doubled the number of trials, we replicated the main findings related to search performance of Experiment 1. Specifically, participants performed better, i.e., had to search fewer trees, in the patchy condition than the dispersed condition and retrieval rates (i.e., number of trees visited to find one fruit item) were better in the patchy than the dispersed condition. Retrieval rates worsened towards the end of each trial when only few fruit items remained. Importantly, and in line with the concept of changing from exploration to exploitation after successful resource encounter in area restricted search (Dorfman et al. 2022) we replicated the finding that the travel distance between trees after unsuccessful tree visits in the patchy condition was longer than when the movement was launched from after a successful tree visit, while there was no difference in the dispersed condition.

The second aim of Experiment 2 was to further investigate the role of (spatial) memory in foraging in our task. Although it is still hotly debated which factor played the greatest role, ecological and social factors together have probably shaped the evolution of brain size in primates (DeCasien et al. 2022; Grabowski et al. 2023). Enhanced foraging efficiency is often mentioned as one ecological benefit of large brains (Grabowski et al. 2023). The idea is that greater cognitive ability improves knowledge and memory of the what (quality and abundance of each resource), where (spatial location), and when (seasonality of food sources) of food sources (Janson 2019; Trapanese et al. 2019; Zuberbühler and Janmaat 2010). Most research into this subject has looked at foraging routes of animals foraging in an area where they know the location of food sources (Hirsch et al. 2024). Hirsch et al. (2024) showed that mammals travelled across their home range with clear understanding of where the best food sources were and that larger brained mammals were not actually foraging more efficiently than smaller brained ones. Less well studied is the role that (short-term) memory plays in assisting animals to avoid revisiting a recently depleted resource (Janson 2019; Hirsch et al. 2024).

To study the role of memory, we introduced the ‘tree-fade’ condition in which trees that had been visited changed their appearance, i.e., they faded out (see Fig. 4). This tree-fading creates a representation of previous visits. Accordingly, participants in the tree-fading condition do not need to memorise which trees have been visited. In addition, the faded trees were less salient than the non-faded trees thus reducing the likelihood that they captured visual attention (Itti and Koch 2000).

In line with this reasoning, participants made fewer errors, i.e., revisited trees less frequently, in the fade condition than in the ‘no fade’ condition. As in Experiment 1, revisits became more frequent later in a trial and retrieval rate dropped, after most of the fruit items had already been collected, and participants searched for the last few fruit items. Importantly, this effect was more pronounced in the ‘no-fade’ condition in which participants had to memorise which trees they had already visited than in the ‘fade’ condition, highlighting the importance of memory for foraging and search tasks in avoiding oversampling (c.f., Kerster et al. 2016; Janson 2019). More revisits in error in the no fade condition is consistent with previous findings in visual search that a memory of several visited locations in a search are maintained albeit with a coarse or low resolution representation (Dickinson and Zelinsky 2007).

Against our predictions, there was no overall difference in foraging performance (neither number of trees visited nor retrieval rate) between the fade and the no-fade condition. We also predicted interactions between tree fading and resource distribution (Bracis et al. 2015) such that tree fading would be particularly beneficial in the dispersed condition. However, we did not observe these interactions for the number of trees visited metric, nor for the retrieval rate metric. So, while tree fading reduced memory errors, it did not significantly affect foraging efficiency. It is possible that the relatively small search space (30 trees) and the use of systematic search strategies such as the lawn mower strategy (see Fig. 3; Tellevik 1992) resulted in too few overall errors to affect search performance. While revisits (i.e., memory errors) did not differ between conditions, revisits increased toward the end of a trial when only few targets remained, and retrieval rate decreased accordingly. This was expected, given that fruits did not replenish within the time of the trial, representative of many food sources (Trapanese et al. 2019), and further highlights the importance of memory in foraging.

Participants searched approximately two trees to find one fruit item in the dispersed condition. This was expected given that 15 fruit items were randomly assigned to 30 trees. As in Experiment 1, retrieval rate in the patchy conditions started off lower than in the dispersed condition but was then higher for the remainder of the trial. We believe this demonstrates a switch from exploration to exploitation behaviour (Dorfman et al. 2022). This switch in search strategy is further evidenced by the inter-tree distance results, i.e., the distance between two successively visited trees, which resembled results from Experiment 1. That is, the travel distance was greater in the patchy than the dispersed condition. This effect was primarily driven by greatly increased inter-tree distance after an unsuccessful visit in the patchy condition. There was also a significant interaction between (1) whether or not the previous tree visit obtained fruit and (2) whether the trial was early or late in the experiment, such that the travel distance following an unsuccessful encounter was greater in the later trials than it was in the earlier trials. We believe that these results are closely related to participants’ foraging strategies and highlights that participants learned the resource distribution in the patchy condition over the course of the experiment. and that they understood that an unsuccessful tree visit in the patchy condition meant they were searching through a part of the environment that did not contain resources and that they needed to explore a different part of the environment. After a successful encounter, in contrast, the inter-tree distance declined which is in line with the idea of area restricted search and a transition to intensive search after resource encounters (Dorfman et al. 2022). Participants’ tendency to return to previously searched locations differed between the patchy and distributed conditions and therefore our paradigm was not sufficiently sensitive to measure and contribute to previously noted and disputed evidence for inhibition of return across search spaces (Klein and MacInnes 1999; Hooge et al. 2005).

## General discussion

In this paper we have introduced a new gaze contingent search task in which participants searched through an environment by looking at items displayed on a computer screen. We demonstrated that participants were sensitive to resource distribution and that they adapted their search/foraging strategy over the course of the experiments accordingly. Specifically, participants performed better, i.e., had to search fewer trees and had higher retrieval rates, when resources were distributed in patches as compared to randomly distributed about the environment. Importantly, and in line with the idea of changing from exploration to exploitation after successful resource encounter in area restricted search (Dorfman et al. 2022) we found that the travel distance between trees after unsuccessful tree visits in the patchy condition was longer than when the movement was launched from after a successful tree visit, while there was no difference in the dispersed condition. This result suggests that participants, once they realised resources were patchily distributed, engaged in exploration behaviour after unsuccessful tree visits. After locating the resource patch, participants then very effectively exploited the patch. We believe that these results demonstrate that our visual search task shares characteristics and cognitive mechanisms involved in successful large-scale search and foraging behaviour (Hills et al. 2015).

The appeal of the visual search task introduced here is (1) that it is a very quick and intuitive laboratory-based foraging task that allows to collect dozens of foraging ‘trajectories’ in a short time period and (2) that it can easily be modified to investigate different and more complex decision-making processes involved in successful foraging behaviour. For example, we are currently running experiments in which participants can collect different types of fruit items that differ in their value and/or in their retrieval time (i.e., reflecting the time individuals invest in item extraction). This allows for the study of the optimal diet model which describes the trade-offs between resource value, search and processing time suggesting that individuals should ignore low profitability resources when more profitable resources are present in abundance (Stephens et al. 2007).

In summary, we tested a number of basic general rules in foraging theory (1) to show the efficacy of our experimental task for testing general theory and (2) to investigate how human foraging strategies follow basic rules used to describe foraging in other animals. Importantly, the (visual) foraging task introduced here can be easily modified in a number of ways (see discussion above) to study how participants integrate knowledge and information about the resources, the distribution of the resources, etc. in their foraging strategies.

## Data Availability

The data and scripts for analyses are available on request from the corresponding author.
